# Thioredoxin 1 and Thioredoxin Reductase 1 Redox System Is Dysregulated in Neutrophils of Subjects with Autism: In Vitro Effects of Environmental Toxicant, Methylmercury

**DOI:** 10.3390/toxics11090739

**Published:** 2023-08-29

**Authors:** Samiyah Alshehri, Sheikh F. Ahmad, Norah A. Albekairi, Sana S. Alqarni, Naif O. Al-Harbi, Laila Y. Al-Ayadhi, Sabry M. Attia, Ali S. Alfardan, Saleh A. Bakheet, Ahmed Nadeem

**Affiliations:** 1 Department of Pharmacology and Toxicology, College of Pharmacy, King Saud University, Riyadh 11451, Saudi Arabia; 2 Department of Medical Laboratory Science, College of Applied Medical Sciences, King Saud University, Riyadh 11451, Saudi Arabia; 3 Department of Physiology, College of Medicine, King Saud University, Riyadh 11451, Saudi Arabia

**Keywords:** autism, thioredoxin 1, methylmercury, neutrophils, oxidative stress

## Abstract

Autism spectrum disorder (ASD) is a complex developmental disorder in children that results in abnormal communicative and verbal behaviors. Exposure to heavy metals plays a significant role in the pathogenesis or progression of ASD. Mercury compounds pose significant risk for the development of ASD as children are more exposed to environmental toxicants. Increased concentration of mercury compounds has been detected in different body fluids/tissues in ASD children, which suggests an association between mercury exposure and ASD. Thioredoxin1 (Trx1) and thioredoxin reductase1 (TrxR1) redox system plays a crucial role in detoxification of oxidants generated in different immune cells. However, the effect of methylmercury and the Nrf2 activator sulforaphane on the Trx1/TrxR1 antioxidant system in neutrophils of ASD subjects has not been studied previously. Therefore, this study examined the effect of methylmercury on Trx1/TrxR1 expression, TrxR activity, nitrotyrosine, and ROS in neutrophils of ASD and TDC subjects. Our study shows that Trx1/TrxR1 protein expression is dysregulated in ASD subjects as compared to the TDC group. Further, methylmercury treatment significantly inhibits the activity of TrxR in both ASD and TDC groups. Inhibition of TrxR by mercury is associated with upregulation of the Trx1 protein in TDC neutrophils but not in ASD neutrophils. Furthermore, ASD neutrophils have exaggerated ROS production after exposure to methylmercury, which is much greater in magnitude than TDC neutrophils. Sulforaphane reversed methylmercury-induced effects on neutrophils through Nrf2-mediated induction of the Trx1/TrxR1 system. These observations suggest that exposure to the environmental toxicant methylmercury may elevate systemic oxidative inflammation due to a dysregulated Trx1/TrxR1 redox system in the neutrophils of ASD subjects, which may play a role in the progression of ASD.

## 1. Introduction

Autism spectrum disorder (ASD) is a complex neurodevelopmental disorder that leads to abnormal social/communication skills and repetitive behavioral patterns/activities [1,2]. ASD is considered to be heritable with complex inheritance and genetic heterogeneity [1]. Recent studies using microarrays, whole-exome sequencing, and whole-genome sequencing have identified that rare de novo mutations as well as variations in single nucleotide polymorphisms are associated with ASD [3,4,5]. However, a consensus is emerging that the total fraction of ASD attributable to genetic inheritance may only be 40–60% [6,7]. Apart from a component of genetic heritability in the pathogenesis of ASD, epigenetic and environmental factors are also known to play an important role [7,8]. Environmental exposure to toxic chemicals has also been implicated in the etiology of ASD [9,10]. Several studies have shown associations between heavy metal environmental exposure and the etiology of ASD. Heavy metals like mercury and lead have been shown to be positively linked to higher incidence/prevalence of ASD [10,11,12,13]. These observations suggest that genetic, epigenetic, and environmental factors in combination produce the phenotype of ASD. However, whether mercury exposure affects an important antioxidant redox system, i.e., Trx1/TrxR1, in the neutrophils of ASD subjects has never been explored earlier.

The thioredoxin (Trx) system is a crucial antioxidant defense mechanism that employs thioredoxin (Trx1), thioredoxin reductase (TrxR1), NADPH to mitigate oxidative stress. This system serves to protect the cells and tissues from excess ROS produced from different oxidative enzymes [14,15,16]. Thioredoxin donates electrons to ROS to detoxify them and in turn it gets oxidized. Oxidized thioredoxin is converted back to reduced form by the help of TrxR1 using NADPH [14]. Further, the TrxR1/Trx1 system serves crucial roles in DNA synthesis and repair through the modulation of ribonucleotide reductase. The Trx1/TrxR1 system also controls many kinases and transcription factors through modulation of redox milieu, ultimately affecting several key intracellular signaling pathways [17].

The Trx1/TrxR1 system is also expressed in cells of immune system such as T cells, B cells, macrophages, and neutrophils. In these immune cells, the Trx1/TrxR1 system has been shown to modulate immune responses to viral and bacterial infections [14,17]. Moreover, the Trx1/TrxR1 system in different immune-mediated diseases has modulated the inflammatory process. For example, Trx has been shown to protect against LPS-induced neutrophil infiltration and cartilage destruction in Trx transgenic through suppression of oxidative stress [18]. In another study, it was shown that Trx-transgenic mice had significantly less neutrophilic inflammation, oxidative damage, and inflammatory markers such as matrix metalloproteases and TNF-alpha, thereby indicating that Trx induction ameliorated cigarette smoke-induced lung inflammation in mice [19]. However, the role of Trx1/TrxR1 in neutrophils with respect to oxidative stress in ASD subjects has not been explored yet.

Neutrophils outnumber other leukocytes in the systemic circulation, showing their importance in the maintenance of a healthy immune system. Neutrophils possess several oxidative enzymes which help in the eradication of invading pathogens during an infection [20]. These enzymes include iNOS, MPO, and NADPH oxidase, which in combination produce several oxidants such as NO, HOCl, ONOO, and superoxide for microbicidal action [21,22]. However, during an inflammatory reaction or chronic illness, neutrophils can cause unwanted oxidative damage using the same oxidative enzymes. Neutrophils play an important role in systemic and neuroinflammation through various mechanisms [20,23]. Neutrophils interact with other immune cells such as T cells, B cells, and macrophages/monocytes. They can modify immune response through NET formation, oxidative burst, and chemokine/cytokine release, which may perpetuate systemic immune imbalance in ASD subjects [20,24]. However, whether oxidative potential is modified by exposure to methylmercury in neutrophils of ASD subjects has not been investigated previously.

ASD subjects have been reported to have dysregulation of different antioxidants in immune cells such as T cells, B cells, monocytes, and neutrophils. Derangements in antioxidants in immune cells include enzymatic antioxidants such as SOD, GPx, GR, HO-1, and antioxidant transcription factors such Nrf2 [25,26,27]. These antioxidants have been studied both in systemic compartment and CNS of ASD subjects. The Trx/TrxR system has also been studied in blood of ASD subjects [28,29,30], however, Trx1/TrxR1 system has not been explored in neutrophils with respect to mercury exposure in ASD subjects.

This study explored whether Trx1/TrxR1 expression and activity was modulated in ASD and TDC subjects. Further, it was explored whether exposure to methylmercury has any effect on the Trx1/TrxR1 system in neutrophils of ASD and TDC subjects. Our study shows that the TrxR1/Trx1 system is dysregulated in neutrophils of ASD subjects, which is linked to increased ROS generation. Further, exposure to methylmercury led to a decrease in TrxR activity and Trx1 expression, and elevation in oxidative potential of neutrophils in ASD subjects. These observations suggest that the Trx1/TrxR1 system in neutrophils of ASD subjects might be prone to inhibition by MeHg exposure, which may enhance overall oxidative stress in the systemic compartment of ASD subjects.

## 2. Materials and Methods

### 2.1. Participants

This cross-sectional study enrolled 29 male children with ASD (age: 7.25 ± 2.87 years, mean ± SD) from the Autism Research and Treatment Center, Faculty of Medicine, King Saud University. If a recruited child had any history of inflammatory/autoimmune disorders (psoriasis, rheumatoid arthritis) or neuropsychiatric/neurological/metabolic illnesses (e.g., phenylketonuria, tuberous sclerosis, mental retardation, depression, seizures), he was excluded from the investigation. The other group consisted of 22 typically developing control (TDC) male children (age: 7.5 ± 2.92 years, mean ± SD) who were enrolled from the Well Baby Clinic, King Khalid University Hospital, Faculty of Medicine, King Saud University, Riyadh, Saudi Arabia. If a TDC child had any history or symptoms pertaining to immune-mediated inflammatory/autoimmune diseases or any intellectual/language disability or any other known genetic diseases, he was not included in the study. Children in both groups were playful, healthy, and active at the time of blood draw through venipuncture and were not given any immune modifying drugs or vitamin supplements. Each child’s parent/guardian gave permission for the involvement of his/her respective child in the study. An approval from the local Ethical Committee of the Faculty of Medicine, King Saud University, Riyadh, Saudi Arabia, was obtained for this study.

### 2.2. Study Measurements

Diagnosis of ASD in every child was confirmed after interviewing the subject. The interview was conducted by a well-trained medical staff and it consisted of subject’s clinical examination, medical history, and neuropsychiatric examination based on the established guidelines for the diagnosis of autism provided in the 5th edition of the Diagnostic and Statistical Manual of Mental Disorders [31]. Moreover, clinical severity of ASD was evaluated based on the Childhood Autism Rating Scale (CARS), which provides a scale of 1–4 in 15 different areas (with a maximum score of 60) for each subject, as detailed before [32]. According to CARS assessment, the ASD group was categorized into two subgroups: mild–moderate category (*n* = 19; M-ASD group) with a score of 30–36; severe category (*n* = 10; S-ASD group) with a score of 37–60.

### 2.3. Separation of Blood Neutrophils

Neutrophils from peripheral blood were separated by density gradient centrifugation, as described earlier [33,34]. Briefly, freshly drawn venous blood in an acid-citrate dextrose Vacutainer tube (BD Biosciences; Franklin Lakes, NJ, USA) was separated into two components using the Ficoll-Paque (1.077 g/mL; Sigma-Aldrich, St. Louis, MO, USA) density gradient centrifugation. Using this technique, blood gets separated into PBMCs (containing T/B cells and monocytes) and a bottom layer mainly consisting of granulocytes. Neutrophils were further separated from the granulocytic cell layer using the dextran sedimentation technique, which provides neutrophils with >95% purity.

### 2.4. Trx Activity Measurement in Neutrophils

Assessment of TrxR activity in isolated blood neutrophils was done through a commercial kit (Cayman Chemical, Ann Arbor, MI, USA). This kit is based on the formation of a colored complex due to enzymatic activity of TrxR which can be measured at 405 nm spectrophotometrically. The formation of colored product is directly proportional to the activity of TrxR in neutrophils. Results are shown as nmol/min/mg protein. 

### 2.5. Flow Cytometry

Blood was taken ASD/TDC subjects and analyzed for different proteins by flow cytometry, as described earlier [26,33]. Neutrophils in blood were first immunolabeled with fluorescently tagged antibody (FITC/APC; Biolegend, San Diego, CA, USA) against extracellular cell surface protein, i.e., CD15. This was followed by the normal steps of permeabilization and fixation of neutrophils for intracellular immunolabeling. Thereafter, neutrophils were immunostained with fluorescently coupled (FITC/APC/PE) antibodies against intracellular proteins, i.e., Nitrotyr, Trx1, TrxR1 (Santa Cruz Biotech, Dallas, TX, USA; Biolegend, San Diego, CA, USA). Samples were run in a flow cytometer (Cytomics FC500 software, Beckman Coulter, Brea, CA, USA) and 10,000 cells were acquired for the evaluation of cell surface marker and intracellular proteins, as described before [26,33].

### 2.6. Neutrophil Cell Culture

Neutrophils were collected from TDC/ASD subjects and incubated under standard conditions in 24-well culture plates with or without methylmercury chloride (0.5 µM final concentration) in RPMI-1640 medium plus heat-inactivated FBS (Invitrogen, MA, USA; Gibco, Grand Island, NY, USA). Further, to assess the effect of the Nrf2 pathway and methylmercury chloride in combination, neutrophils were incubated with sulforaphane (5 µM final concentration) for 30 min before addition of methylmercury chloride. Neutrophils were incubated for 12 h in standard culture conditions for TrxR activity, Trx1/TrxR1 expression, Nrf2 assay, and oxidative stress measurements.

### 2.7. Nrf2 Trans-Activation ELISA Assay in the Neutrophils

Evaluation of Nrf2 trans-activation binding potential to antioxidant response element (ARE) in nuclear extracts of neutrophils was conducted through a commercially available Trans-AM Nrf2 kit (Active Motif, Carlsbad, CA, USA). This ELISA assay uses the principle of binding of ARE, i.e., 5′-GTCACAGTACTCAGCAGAATCTG-3′, present in the plate to the Nrf2 extracted from the sample. Binding of Nrf2-ARE leads to color formation, which can be measured at 450 nm in a microplate spectrophotometer. Formation of colored complex in ELISA reaction is directly proportional to the specific activity of the Nrf2 in the neutrophilic nuclear extracts. Data are displayed in fold difference.

### 2.8. Data Analysis

The data are shown as mean ± SEM. Comparison of different biochemical parameters among different groups was analyzed by one-way ANOVA (analysis of variance) followed by multiple comparison post hoc test, i.e., Tukey’s test. The data of different parameters were normally distributed as assessed by Shapiro–Wilk test. The level of statistical significance was set at *p* < 0.05. All the statistical analyses were performed using the Graph Pad Prism 9 statistical package (GraphPad Software, San Diego, CA, USA).

## 3. Results

### 3.1. TrxR1 Expression and Trx Activity in ASD and TDC Neutrophils

**Figure 1:**
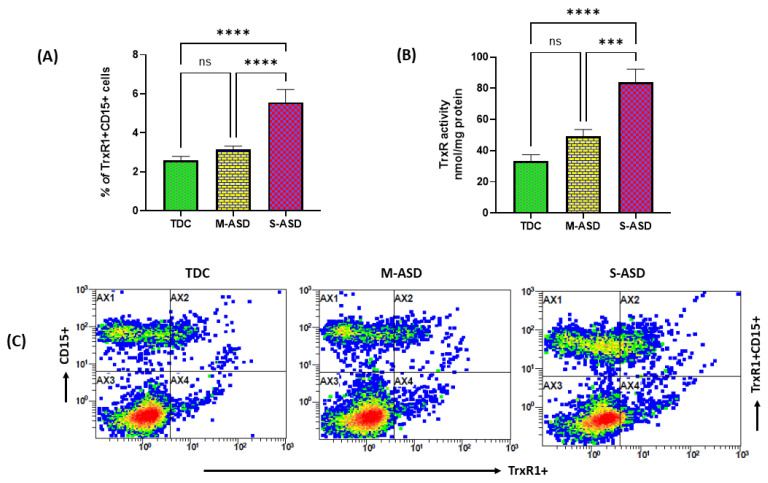
Expression of TrxR1 and TrxR activity in TDC/ASD neutrophils. (**A**) % of TrxR1+CD15+ cells, (**B**) TrxR enzymatic activity, and (**C**) representative flow plot displaying TrxR1+CD15+ double immunostaining. Values are shown as mean ± SEM, n = 10–22/group. *** *p* < 0.001; **** *p* < 0.0001; ns = not significant.

The TrxR1/Trx1 redox system is known to regulate oxidant–antioxidant balance in different immune cells, however, its role has not been explored previously in neutrophils of ASD and TDC subjects. Therefore, we first measured the protein expression of TrxR1 in neutrophils of both groups. Our data show that TrxR1 protein expression is increased in ASD neutrophils as compared to TDC neutrophils, as displayed by increased % of TrxR1+CD15+ neutrophils in systemic circulation (Figure 1). Further, when classified according to disease severity, the severe ASD group had further elevation in TrxR1 protein expression as compared to the mild/moderate ASD group (Figure 1). TrxR activity was also measured in both groups. TrxR enzymatic in neutrophils was significantly increased in ASD group as compared to the TDC group. Further, the severe ASD group had significantly higher neutrophilic TrxR activity than the mild/moderate ASD group (Figure 1). These data suggest that TrxR1/TrxR activity is increased, which may be a compensatory response to mitigate increased oxidant stress in the ASD group.

### 3.2. Trx1 Expression in ASD and TDC Neutrophils

**Figure 2:**
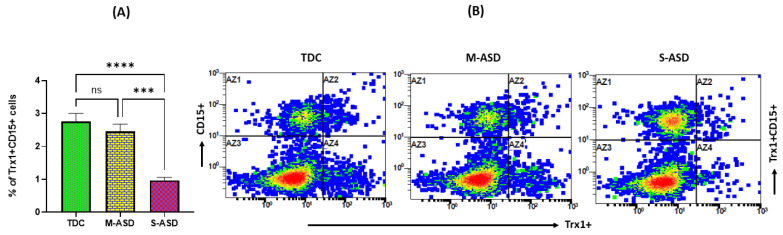
Expression of Trx1 in TDC/ASD neutrophils. (**A**) % of Trx1+CD15+ cells, and (**B**) representative flow plot displaying Trx1+CD15+ double immunostaining. Values are shown as mean ± SEM, n = 10–22/group. *** *p* < 0.001; **** *p* < 0.0001; ns = not significant.

Next, the substrate for TrxR1, i.e., Trx1, was also measured in the neutrophils of both groups as it has antioxidant functions alone and in combination with TrxR1. Our results display that Trx1 protein expression is decreased in ASD neutrophils as compared to TDC neutrophils, as displayed by decreased % of Trx1+CD15+ neutrophils in systemic circulation (Figure 2). Further, when classified according to disease severity, the severe ASD group had a further decrease in Trx1 protein expression as compared to the mild/moderate ASD group (Figure 2). These data suggest that the TrxR1/Trx1 redox couple is dysregulated in the neutrophils of the ASD group.

### 3.3. Effect of MethylMercury on TrxR/Trx Redox Couple in ASD and TDC Neutrophils

**Figure 3:**
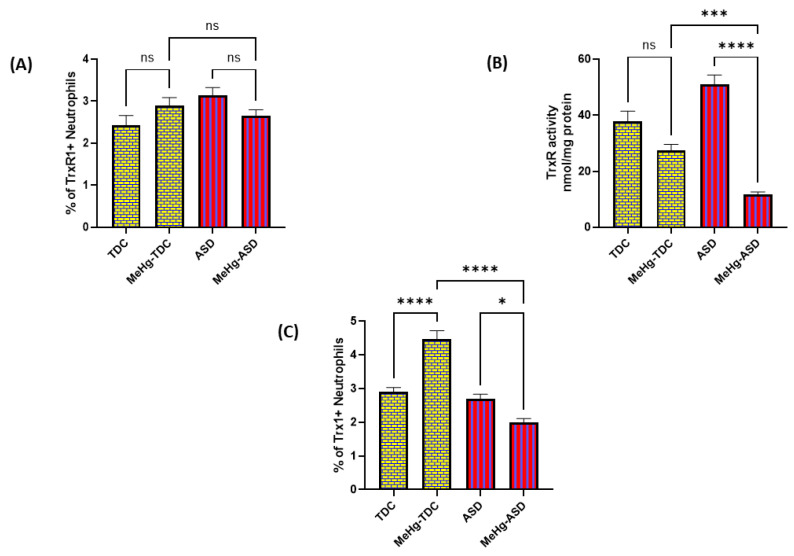
Effect of methylmercury on the expression of TrxR1 and activity of TrxR in TDC/ASD neutrophils. (**A**) % of TrxR1+neutrophils, (**B**) TrxR enzymatic activity, and (**C**) % of Trx1+Neutrophils. Values are shown as mean ± SEM, n = 14/group. * *p* < 0.05; *** *p* < 0.001; **** *p* < 0.00001; ns = not significant.

Children with ASD may be exposed to mercury compounds through environmental exposure, therefore, we next wanted to explore whether methylmercury had any effect on the TrxR1/Trx1 redox couple in ASD and TDC neutrophils. Our results depict that TrxR1 protein expression is unaffected in ASD neutrophils after methylmercury treatment whereas TDC neutrophils show an increase in TrxR1 expression which is not significant, as displayed by decreased % of TrxR1+CD15+ neutrophils (Figure 3A). TrxR activity was also measured in both groups with and without methylmercury treatment. TrxR enzymatic in neutrophils was significantly decreased in both groups, however, the magnitude of inhibition was much greater in the ASD neutrophils (Figure 3B). Next, the Trx1 protein expression was also measured in neutrophils of both groups with and without methylmercury treatment. Our results display that Trx1 protein expression was decreased in ASD neutrophils whereas TDC neutrophils had increased Trx1 protein expression after treatment with methylmercury, as displayed by % of Trx1+CD15+ neutrophils (Figure 3C). These data suggest that the TrxR1/Trx1 redox couple is inhibited to a greater extent by methylmercury in ASD neutrophils than TDC neutrophils.

### 3.4. Effect of MethylMercury on Oxidative Stress Markers in ASD and TDC Neutrophils

**Figure 4:**
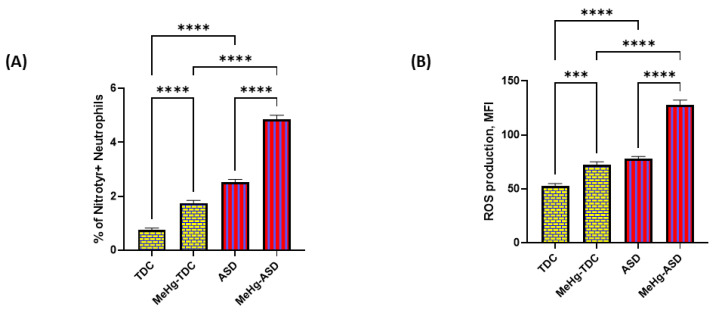
Effect of methylmercury on ROS/nitrotyrosine levels in TDC/ASD neutrophils. (**A**) % of nitrotyrosine+neutrophils, (**B**) MFI for ROS generation. Values are shown as mean ± SEM, n = 14/group. *** *p* < 0.001; **** *p* < 0.0001.

As the Trx1/TrxR1 redox couple is the gatekeeper of oxidant–antioxidant balance in various cells, we next explored if a reduction in Trx1/TrxR1 expression had any effect on oxidative inflammation in neutrophils of the ASD and TDC groups. Our results show that nitrotyrosine immunostaining is significantly increased in ASD neutrophils compared to TDC neutrophils after treatment with methylmercury (Figure 4A). Next, overall ROS generation was also measured, which showed a pattern like nitrotyrosine. ROS generation as shown by MFI was much greater in ASD neutrophils than TDC neutrophils after treatment with methylmercury (Figure 4B). These data suggest that inhibition of the TrxR/Trx redox couple by methylmercury causes exaggerated oxidative inflammation in ASD neutrophils.

### 3.5. Effect of Sulforaphane on MethylMercury-Induced Changes in Trx1/TrxR1 System in ASD and TDC Neutrophils

**Figure 5:**
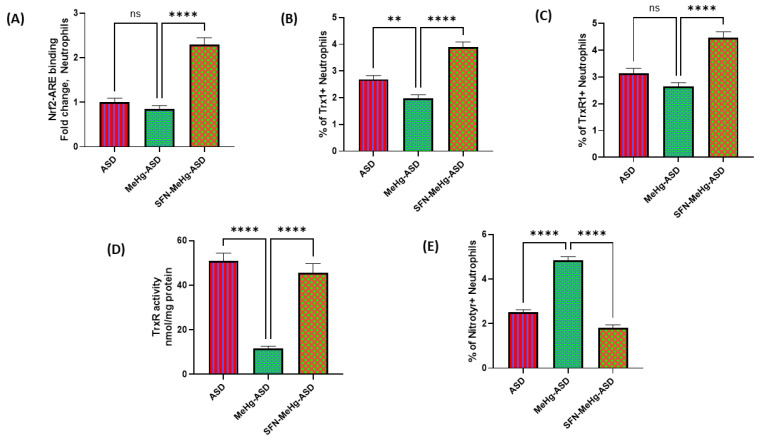
Effect of the Nrf2 activator sulforaphane on methylmercury-induced changes in the Trx1/TrxR1 system and nitrotyrosine levels in TDC/ASD neutrophils. (**A**) Nrf2 binding assay, (**B**) % of Trx1+Neutrophils, (**C**) % of TrxR1+Neutrophils, (**D**) TrxR1 activity, and (**E**) % of nitrotyrosine+neutrophils. Values are shown as mean ± SEM, n = 14/group. ** *p* < 0.01; **** *p* < 0.0001; ns = not significant.

Next, we wanted to delineate whether a lack of antioxidant upregulation in ASD neutrophils was associated with Nrf2 signaling. For this purpose, we used the Nrf2 activator sulforaphane before incubation with methylmercury in ASD neutrophils. Our results show that sulforaphane led to increased ARE binding, as depicted by increased translocation of Nrf2 to the nucleus in ASD neutrophils (Figure 5A). This was associated with elevated levels of Trx1/TrxR1 antioxidant proteins and TrxR activity (Figure 5B–D). Furthermore, methylmercury-induced nitrotyrosine immunostaining was significantly downregulated in ASD neutrophils by sulforaphane pretreatment (Figure 5E). These data suggest that a defective Trx1/TrxR1 system can be corrected by activation of Nrf2 signaling in methylmercury exposed neutrophils in ASD subjects.

## 4. Discussion

The Trx1/TrxR1 system plays a strong protective role against oxidative/nitrosative stress induced by several agents. TrxR1 is a selenocysteine disulfide oxidoreductase enzyme and is abundantly expressed in the cytosol of mammalian cells. Its main function is providing defense against various oxidative insults. TrxR1 may catalyze the reduction of H_2_O_2_, lipid hydroperoxides, and dehydroascorbate through its natural substrate, Trx1 [14,15]. The reduction of oxidized Trx1 is catalyzed by TrxR1 using NADPH. Trx-1 is a small, 12-kDa, ubiquitous protein with 2 redox-active cysteine residues (Cys32–Cys35) in the active center, which is essential for its redox regulatory function [35]. Our study showed a disturbance in TrxR1/Trx1 redox system in neutrophils of ASD subjects for the first time.

In addition to its potent anti-oxidative effect, Trx1/TrxR1 system also has anti-inflammatory properties, mainly because of its ability to inhibit neutrophil chemotaxis to inflammatory sites and to suppress the expression and activation of other inflammatory factors [35,36]. The TrxR1/Trx1 system is important for regulation of cellular redox milieu, which in turn may regulate energy metabolism, apoptosis, cell growth, differentiation, angiogenesis, immune responses, antioxidant defenses, and systemic/neuroinflammatory processes [36]. Therefore, dysfunctions in the TrxR1/Trx1 redox couple are associated with various inflammatory/oxidative disorders [19,35,37]. Our study also showed that dysregulation in the TrxR1/Trx1 redox couple was associated with increased oxidative inflammation in neutrophils of ASD subjects.

There is evidence of systemic immune-oxidative inflammation in ASD which includes: (1) activated immune cells (monocytes/neutrophils/T cells/B cells); (2) increased oxidative stress (e.g., elevated 3-nitrotyrosine, myeloperoxidase, oxidized glutathione levels, increased lipid peroxides); (3) elevated 8-oxo-guanosine levels (oxidative damage marker of DNA); (4) elevated chemokines/cytokines (IL-6, IL-17A, IFN-gamma); (5) elevated expression of proinflammatory transcription factors (NF-kB, STAT3) [38,39,40,41,42]. Similar changes have been shown to occur in the brain of ASD subjects, suggesting that immune cells of the periphery and CNS communicate with each other and may equally contribute to the oxidative inflammation observed in ASD subjects [1,27,43]. Our study showed that levels of 3-nitrotyrosine and ROS were higher in ASD neutrophils than TDC neutrophils, which confirms earlier reports. 

Neutrophils are professional phagocytes that play a central role in the innate immune system [44]. For microbicidal action, neutrophils produce several oxidants including hydrogen peroxide, hypochlorous acid, and chloramines [45,46]. Sustained elevation in ROS during chronic immune-mediated disorders can induce damage to DNA, proteins, and lipids, thereby leading to irreversible nitration of functional enzymes and alteration in bilipid membrane structures, which may lead to pathological consequences [24]. This may happen due to consumption or oxidation of intracellular antioxidants in neutrophils [47]. Thus, to protect the interior of the neutrophil from excessive oxidants, an efficient antioxidant defense machinery is required so that cells can function properly. A major component of cellular antioxidant defense in neutrophils is the TrxR1/Trx1 redox system. This system scavenges cellular peroxides and other ROS, thereby maintaining redox balance of the cell [24,45,48]. Our study showed a dysregulated TrxR1/Trx1 system which could lead to increased oxidative inflammation in ASD neutrophils, as observed in this study. 

Mercury is a ubiquitous environmental toxicant with recognized adverse health effects throughout the world. Humans are exposed to mercury compounds through industrial waste, agriculture, and medicine. Several studies consider mercury as a risk factor for the development of ASD [1,49]. These studies show that ASD subjects are more vulnerable to mercury compounds than TDC subjects due to the presence of higher levels of mercury compounds in different body tissues such as nails, hair, blood, and urine in the former group. Further, levels of mercury in the body tissues correlate with the severity of symptoms [1,11,12]. Mercury compounds may cause neurodevelopmental and systemic changes through different mechanisms, which include inactivation of antioxidants, oxidative stress, neuronal damage, and loss of neuronal connectivity [1,49]. Our study shows that methylmercury inactivates the TrxR/Trx1 redox system in ASD neutrophils that is associated with exaggerated oxidative inflammation.

The Trx1/TrxR1 system has been shown to be particularly sensitive to mercury compounds. TrxR1 is a prime target for methylmercury due to its high reactivity with selenocysteine residues located in the enzyme [49,50]. Several in vitro and in vivo investigations have reported a significant inhibition of TrxR activity in liver and brain tissues after exposure to different mercury compounds [51,52]. Our study also showed decreased TrxR activity in ASD and TDC neutrophils upon mercury treatment, however, it was significant only in the ASD group. Further, decreased TrxR activity was associated with elevated Trx1 protein expression in TDC neutrophils but not ASD neutrophils. In liver cells, microglia and neuronal cell inhibition of TxR1 by mercury compounds led to elevation of Trx1/TrxR1 proteins via Nrf2-mediated transcriptional upregulation [50,53,54]. This may be a strategy of TDC neutrophils to counteract elevated oxidative stress caused by mercury exposure, whereas ASD neutrophils may face defective antioxidant mechanisms, thereby not allowing them to sufficiently scavenge ROS, as depicted by a marked increase in markers of oxidative stress.

In the context of an association of heavy metal exposure such as mercury with the etiology of ASD, it is noteworthy that heavy metal exposure by itself might not be the sole contributing factor in this phenomenon, the body’s ability to eliminate the environmental toxins could also play a key role. It is plausible that mercury toxicity may be related to a decreased ability of ASD children to detoxify this heavy metal rather than to the level of exposure. This observation suggests that detoxification mechanisms may be altered in ASD subjects, which may predispose them to neuro-immune dysregulation [13,55]. However, future studies are needed to explore such a hypothesis.

Cellular redox status in terms of antioxidant system and/or oxidative stress may affect the function of neutrophils, thereby leading to detrimental inflammatory effects. Attachment of neutrophils to the vascular endothelium is enhanced by vascular oxidative stress that may play a key role in the neuroinflammatory process [22]. Increased ROS is linked to upregulated chemokine receptors such as CXCR2 on the neutrophil membrane, which may increase transendothelial migration of neutrophils into various tissues, including CNS, thereby leading to neuroinflammation [56,57]. Systemic oxidative stress caused by neutrophilic oxidative enzymes has been reported to cause microvascular dysfunction, neuroinflammation, and memory deficits in animal models of neurological diseases recently [20,58]. Overactivation of neutrophils has also been shown to be involved in induction of neurovascular inflammation during autism [23]. Increased ROS generated within methylmercury-treated neutrophils in ASD subjects may cause aggravated oxidative damage to other immune cells, vascular cells, and at the junction of BBB, thereby promoting systemic/neuroinflammatory process. 

The TrxR1/Trx1 redox system is known to regulate Nrf2 signaling in mammalian cells. All cells exhibit coordinated antioxidant protection in response to stressful stimuli such as mercury compounds, which is mainly regulated by ubiquitous transcription factor Nrf2 [24,52,59]. After activation by different types of stress signals, Nrf2 moves to the nucleus and binds to the antioxidant responsive element (ARE) in the promoter region of genes that regulate antioxidant enzymes controlling redox equilibrium such as Trx1, TrxR1, SOD, and HO-1 [26]. It has also been shown that the TrxR1/Trx1 system controls Keap1 (Kelch-like ECH-associated protein 1), that is the main switch for activation of Nrf2 signaling during stressful situations [52]. It was reported that mice lacking TrxR1 (Txnrd1 deficient gene) had increased accumulation of nuclear Nrf2 protein which contributed to an efficient antioxidant protective response in the liver [60]. Activation of Nrf2 due to mercury-induced inhibition of the TrxR1/Trx1 system may be required to counteract amplification of oxidative stress [59,61]. However, after exposure to methylmercury in our study, neutrophils in ASD subjects did not have elevated Trx1 levels whereas TDC neutrophils had increased Trx1 protein expression. This could be due to defective Nrf2 signaling in ASD neutrophils. Previous reports have shown that ASD subjects have impairment in the translocation of Nrf2 from the cytoplasm to the nucleus, thus leading to diminished antioxidant response [26,40,62]. This was confirmed by using the Nrf2 activator sulforaphane, which achieved upregulation of Nrf2-mediated proteins such as Trx1 and TrxR1 with concomitant reduction in oxidative stress in ASD neutrophils. These observations suggest that dysregulation in the Trx1/TrxR1 system induced by methylmercury can be corrected by the Nrf2 activator compound sulforaphane.

Sulforaphane has been shown to ameliorate autistic behavior in human subjects. ASD subjects treated with sulforaphane showed improvement in autism-related behaviors such as social interaction and verbal communication [63,64,65]. However, a recent study showed no significant clinical improvement in the behavioral outcomes in ASD subjects after treatment with sulforaphane [66]. Different outcomes in these studies could be due to a difference in study designs and the genetic/environmental backgrounds of ASD subjects. More studies are needed to ascertain the role of in vivo administration of sulforaphane in behavioral and cognitive functions in ASD subjects.

## 5. Conclusions

Overall, our study suggests that the Trx1/TrxR1 redox system is dysregulated in ASD neutrophils. Further, methylmercury exposure fails to elevate the Trx1/TrxR1 redox couple in ASD neutrophils, which is associated with exaggerated oxidative inflammation. These observations suggest that environmental exposure to methylmercury may detrimentally increase systemic oxidative inflammation in ASD subjects.

## Data Availability

The authors confirm that all data underlying the findings are fully available without restriction. All relevant data are within the paper.
